# Understanding alcohol use around water: A qualitative analysis of the experiences of young males

**DOI:** 10.1111/bjhp.12809

**Published:** 2025-06-23

**Authors:** Sabryna V. Sas, Jacob J. Keech, Amy E. Peden, Martin S. Hagger, Kyra Hamilton

**Affiliations:** ^1^ School of Applied Psychology Griffith University Brisbane Queensland Australia; ^2^ School of Population Health, Faculty of Medicine and Health University of New South Wales Sydney New South Wales Australia; ^3^ College of Public Health, Medical and Veterinary Sciences James Cook University Townsville Queensland Australia; ^4^ Health Sciences Research Institute University of California, Merced Merced California USA; ^5^ Psychological Sciences University of California, Merced Merced California USA; ^6^ Faculty of Sport and Health Sciences University of Jyväskylä Jyväskylä Finland

**Keywords:** alcohol, drowning, injury, males, social cognition beliefs, swimming, theory of planned behaviour

## Abstract

**Objectives:**

Alcohol is a significant risk factor for drowning, with young males disproportionately impacted. We explored salient social cognition beliefs that influence young Australian males' consumption of alcohol around water.

**Design:**

Via purposive sampling, 23 Australian males aged 18–30 years who had previously consumed alcohol around water were recruited. A brief online survey gathered demographics, eligibility criteria and swimming ability, followed by a qualitative interview via telephone. An interview guide based on established methods for exploring social cognition factors gathered data on behavioural, normative and control beliefs. Interviews were audio‐recorded, transcribed verbatim and coded using theoretical thematic analysis in NVivo.

**Results:**

Advantages of consuming alcohol around water included enjoyment, fun and relaxation (73%), and alcohol as ‘liquid courage’ (21%). Disadvantages included increasing the risk of drowning and injury (100%), and inhibiting judgement (73%). Normative beliefs included family approval (52%) and disapproval (86%), in addition to approval from friends (82%) and disapproval from lifeguards (56%). Control beliefs included access to alcohol (52%), confidence derived from knowledge about the body of water (26%) and legality (26%). The presence of signage, authority figures or children and families helped to prevent young males from consuming alcohol.

**Conclusions:**

Results indicate respondents drink and swim despite reporting an awareness of the risks associated. Intervention strategies that aim to address behavioural, normative and control beliefs of young males as well as contextual factors supporting or hindering the behaviour may help to curtail alcohol misuse around water, particularly when targeted during warmer weather and holiday periods.

**Statement of contribution**

**What is already known on this subject?**

- Drowning is a neglected public health issue, with males significantly overrepresented.
- Alcohol is a key risk factor for drowning, particularly among young males.
- Few evaluated interventions exist, particularly those underpinned by behavioral theory.

**What does this study add?**

- Fun, liquid courage and encouragement were reasons why young males consume alcohol around water.
- Disapproval, signage and presence of authority and children discourage alcohol use around water.
- Insights can guide future interventions designed to prevent this risky aquatic activity.

## INTRODUCTION

Drowning is a preventable public health threat which accounts for 7% of all injury‐related deaths, making drowning the third leading cause of injury‐related deaths worldwide (World Health Organization, 2014). Males continue to be overrepresented in drowning statistics (Croft & Button, 2015; Howland et al., 1996; Peden et al., 2021) with alcohol and drug use significant risk factors for drowning among young males (Hamilton, Keech, et al., 2018; Peden et al., 2018). Yet, there is limited quality research investigating prevention efforts for reducing alcohol use around water, with just three studies identified in the past two decades (Hamilton, Keech, et al., 2018). Also, limited empirical literature has examined the subjective experiences of males and their knowledge, beliefs and attitudes towards alcohol consumption around water. In the few studies available, findings indicate that previous experience, peer presence, social acceptability and social situations increase the likelihood of alcohol consumption, despite individuals knowing the risks (Abercromby et al., 2020; Calverley et al., 2020, 2021; Hamilton & Schmidt, 2014; Leavy et al., 2022). Given drownings from alcohol are often preventable and commonly occur among males, it is important to identify the beliefs young males hold regarding this important water safety behaviour, which can then serve as a useful source of strategies to target in future interventions.

As a belief‐driven theory widely used in health and social psychology (McEachan et al., 2011), the theory of planned behaviour (TPB) (Ajzen, 1991) posits intention as the proximal predictor of behaviour with attitude (positive and negative evaluations of the behaviour), subjective norm (perceived pressure from significant others) and perceived behavioural control (perceived confidence and control) predicting intention, and in turn predicting behaviour. Further, the model proposes that the attitude, subjective norm and perceived behavioural control constructs are underpinned by sets of more specific salient behavioural (advantages/disadvantages), normative (approval/disapproval from specific referents) and control beliefs (facilitators/barriers), respectively (Ajzen, 1991). Elicitation of these beliefs is considered a strength of the TPB, as the global measures of attitude, subjective norm and perceived behavioural control, which are often tested as antecedents of intention and behaviour, are merely summative states of more fundamental lower‐level elements (i.e., beliefs) and, therefore, the action of behaviour change tends to be at the belief, rather than summative, level. The optimal point for changing the global constructs, therefore, is through directly targeting the underpinning beliefs.

The TPB has been used successfully as the basis of prediction for many studies of health (Hagger & Hamilton, 2024; Hagger et al., 2002, 2022; Hamilton, Smith, et al., 2022; McEachan et al., 2011) and drowning prevention (Hamilton et al., 2019; Hamilton et al., 2023) behaviours. Furthermore, the TPB belief‐based approach, as a theoretical basis for the creation of health messages, has proved successful in changing health behaviour (Steinmetz et al., 2016), including in behaviours for being safe around water (Hamilton, Keech, et al., 2018, 2022; Hamilton, Peden, et al., 2018; Hamilton et al., 2021). Adopting a theoretical approach to identifying salient beliefs can provide critical targets for interventions tailored to a specific behaviour, population group or context (Ajzen & Schmidt, 2020). The use of the TPB as a framework to explore young males' beliefs underpinning their decisions to consume alcohol around water may, therefore, provide the basis to create theoretically based and empirically driven messages that can inform future interventions to increase the likelihood of the intervention being efficacious (Hamilton, Keech, et al., 2018, 2022; Hamilton, Peden, et al., 2018; Hamilton et al., 2021; Steinmetz et al., 2016).

The aim of the current study was to identify the salient beliefs young males hold towards consuming alcohol around water, based on the perspectives and experiences of males who have previously engaged in this behaviour. We adopted a TPB belief‐based approach to identify behavioural, normative and control beliefs for the target behaviour and population, young males. Identified beliefs can be further tested to determine their efficacy in changing behaviour and, thus, assist in the design of future interventions to promote responsible drinking of alcohol around water, ultimately preventing drownings and saving lives.

## METHOD

### Participants

Participants were 23 males with experience of having engaged in the target behaviour and who resided in Australia. Ages ranged from 18 to 30 years (mean age = 23 years; SD = 3.43). Approximately half of the sample were university students (56%). Most participants came from an English speaking background (78%), with a fifth of the sample reporting coming from a non‐English speaking background (21%). See Table 1 for detailed demographic information.

**TABLE 1:** Participant demographic characteristics.

Demographic characteristic	Participants (*N* = 23)
Marital status	
Married registered	0
Married de facto	1
Separated/divorced	0
Widowed	0
Never married	22
Non‐English speaking background	
Yes	5
No	18
Employment status	
Full‐time work	3
Part‐time/casual work	8
Full‐time student	10
Part‐time student	3
Unemployed/home duties	5
Household income (annual)	
Nil–$18,200	8
$18,201–$37,000	3
$37,001–$80,000	8
$80,001–$180,000	4
>$180,001	0
Highest educational attainment	
Completed junior school (year 10)	0
Completed senior school (year 12)	7
TAFE certificate/diploma	3
Undergraduate degree	11
Postgraduate degree	2
Age	
Mean (SD)	23.43 (3.44)
Range	18–30
Current Swimming ability	
1 (poor)	0
2	0
3	0
4	2
5	9
6	6
7 (Excellent)	6
Achievable length of consistent swimming without stopping	
1 (cannot swim)	0
2 (less than 25 m)	0
3 (25 up to 100 m)	6
4 (100 up to 200 m)	4
5 (200 up to 300 m)	5
6 (300 up to 400 m)	0
7 (more than 400 m)	8

### Design and procedure

This project received ethical approval from the University Human Research Ethics Committee (ref no: 2019/294). A purposive sampling method was used to recruit Australian adult males who met the experience criteria of having consumed alcohol in and around water. Individuals were recruited using social media posts, online media releases and university broadcast emails. Participants received an AU$50 department store gift card upon completion of the interview. No participants dropped out after agreeing to participate. Data were collected throughout September and October 2020. The study was guided by the COREQ (consolidated criteria for reporting qualitative research) checklist (Tong et al., 2007).

Participants were given an information sheet explaining the study. After providing verbal consent, participants completed a brief online demographic survey lasting approximately 5 min, which collected information on participants' age, gender, marital status, highest level of education, household income and current swimming ability. This was followed by a semi‐structured interview conducted via telephone that contained a range of open‐ended questions. Only the researcher was present at their location when conducting the interview, and participants were encouraged to do the interview in a private and quiet place. The interviews lasted approximately 30 min. Interviews were conducted by the first author who holds university qualifications in psychology and criminology. At the time of the study, the first author was a research assistant and PhD candidate. The interviewer had no relationship with participants prior to the interviews, and participants were made aware of the interviewer's role and the goal of the study prior to commencing the interview. The interviewer was trained in qualitative interview techniques and had extensive experience in roles requiring the ability to effectively build rapport with people from diverse backgrounds. The interviewer leveraged this experience to enable her to adopt a stance of genuine curiosity and interest, creating a conversational tone that allowed participants to speak openly about their experiences without fear of judgement. Building rapport was prioritized through active listening, empathic engagement and the use of informal language where appropriate to signal approachability and respect. Reflexivity was an ongoing process. The interviewer maintained a reflexive journal to document personal reflections and assumptions that emerged during data collection and analysis. Awareness of potential biases, such as normative beliefs about alcohol use and risk‐taking, was central to maintaining a critical lens on how data were interpreted. Peer debriefing and consultations with the research team were used to discuss interpretations and assumptions, supporting the rigour and trustworthiness of the analysis.

Participants were asked to openly share their experience of consuming alcohol around water, along with their beliefs that facilitated this decision. The interviewer used techniques such as confirming summaries to check understanding and minimize bias and assumptions (Conte, 2009). Interviews were conducted until all participants who had expressed interest and were available to be interviewed had completed an interview. Interviews were audio‐recorded and transcribed verbatim (removing any identifying data and assigning pseudonyms). Data from interview transcripts were imported into NVivo software (Version 11.0) to facilitate coding using theoretical thematic analysis (Braun & Clarke, 2006). The first author coded the data and extracted initial themes. Themes were refined through discussion among the research team. The first author re‐reviewed transcripts to ensure themes were consistent with the original context in which they were described.

### Interview guide

The target behaviour of interest was *alcohol consumption around water*. To explore this specific behaviour, an interview protocol was developed to elicit beliefs underpinning decisions to consume alcohol around water. The interview questions were developed by researchers and industry professionals with expertise in behavioural science and drowning prevention, and the interview guide was pilot tested prior to use, resulting in minor edits being made to question wording to improve clarity. Questions were based on established methods for eliciting social cognition beliefs based on the theory of planned behaviour (Ajzen, 2006) and prior research by the research team (Hamilton et al., 2019; Hamilton, Keech, et al., 2018; Hamilton, Peden, et al., 2018).

#### Behavioural beliefs

Behavioural beliefs were elicited with two open‐ended questions regarding the advantages and disadvantages of drinking alcohol around water (e.g., ‘What do you see as the advantages/disadvantages of drinking alcohol around water?’).

#### Normative beliefs

Normative beliefs were elicited with two open‐ended questions regarding who would approve and disapprove of the young male drinking alcohol around water (e.g., ‘Who are the individuals or groups of people who would approve/disapprove of you drinking alcohol around water?’).

#### Control beliefs

Control beliefs were elicited with two open‐ended questions about the barriers and facilitators for drinking alcohol around water (e.g., ‘What are the factors that make it easy/difficult for you to drink alcohol around water?’).

### Data analysis

The data were analysed using theoretical thematic analysis, with the assistance of NVivo qualitative analysis software (Braun & Clarke, 2006, 2013; Joffe & Yardley, 2004). To ensure a rigorous and methodologically sound approach, the analysis followed Braun and Clarke's (2006) six‐phase process. First, transcripts were repeatedly read to allow the first author to become deeply familiar with the data, during which notes on potential themes and interesting observations were made. Familiarization was strengthened by the first author conducting the interviews herself. Following this stage, initial coding was conducted for each interview transcript. Similar codes were grouped into preliminary themes, which were iteratively developed and reviewed. Broader themes were identified and organized based on theory and the frequency of codes across the interviews. These themes were then merged where appropriate, refined and given specific definitions and labels before being written up. The first author performed the initial coding of the data. Codes and themes were refined through discussion between the first, second, and last author to ensure consistency and accuracy. Final themes were reviewed by all authors.

## RESULTS

Various themes were identified in the males' descriptions about their decisions to consume alcohol around water. Themes were organized based on the guiding theoretical TPB belief‐based approach of eliciting behavioural, normative and control beliefs. The most salient themes across participants are presented below. These themes were identified in at least 10% of participants. Extracts are indicated by participant number (e.g., P01). Please see Table 2 for a summary of the themes.

**TABLE 2:** Summary of themes.

TPB beliefs		Themes
Behavioural beliefs	Advantages of drinking alcohol around water	A way to cool down on a hot day
		It is enjoyable, fun and relaxing
		Helps me to hang out with friends
		“Liquid courage”
	Disadvantages of drinking alcohol around water	Makes me feel unwell during and after
		Environmental impact
		It increases the risk of drowning and injury
		Inhibits my judgement
		Can lead to legal punishment
		Risk to one's reputation
Normative beliefs	Approval of drinking alcohol around water	Approval from family
		Approval from friends
		Police would not mind
	Disapproval of drinking alcohol around water	Family disapproval
		Health and emergency services
		Lifeguards
		Police
Control beliefs	Facilitators of drinking alcohol around water	Having access to alcohol
		If the alcohol is not visible to authorities
		If it is a celebration and I am spending time with others
		Knowledge of water conditions gives confidence to drink
		It's legal
		If I was pressured
		If I saw other people doing it
		Warm weather
	Barriers to drinking alcohol around water	If the alcohol is difficult to access
		If I was alone
		If it wasn't safe
		If laws and signs were clear
		Having an ‘official’ presence around
		If you need to drive
		If I need to supervise kids
		If I had something else on that day
		If there were kids and families present

### Behavioural beliefs

#### Advantages of drinking alcohol around water

##### A way to cool down on a hot day

Just over a quarter of the participants (26%) described the effect of the alcohol and water cooling them down on a hot day as a key advantage of engaging in the behaviour. For example, one participant reported, ‘A lot of the times when I do it, it is in the warmer weather, so it's just nice to cool off’.—*P19*.

##### Makes it enjoyable, fun and relaxing

Many participants (73%) described experiences of enjoyment, fun and relaxation that are associated with alcohol consumption around water. One participant reported that ‘Oh it's… quite relaxing to be in the water whilst intoxicated. Like especially at the beach at night‐time and it's quite a good, yeah it feels nice in the water’.—*P14*. Another participant described ‘It's fun. But I mean doing anything while drinking alcohol is fun, that's why we drink alcohol, to have fun’.—*P12*.

##### Helps me to hang out with friends

Several participants reported that alcohol consumption around water assisted them in socializing and hanging out with friends (34%). For example, ‘You know…drinking is social. Might be a good way to connect with friends or something similar and if everyone's around the water’.—*P05*.

##### ‘Liquid courage’

Participants also described the effects of alcohol consumption in increasing their confidence and courage in social situations as an advantage (21%). One participant described the effect as ‘liquid courage’. They stated ‘personally I can see how there is one benefit, it's because like I speak multiple languages… obviously you've heard the term like liquid courage. So, when I drink you know like my language abilities are so much better. Because I don't really think about like what people think and I just say whatever is on my mind…’—P16. Another participant described reduced fear and anxiety around water following alcohol consumption, ‘Oh probably less fear or less anxiety about it? Yeah, like if someone doesn't normally like to swim or anything, they might not think about it as much’.—*P22*.

#### Disadvantages of drinking alcohol around water

##### Makes me feel unwell during and after

Several participants described that physical sensations of sickness or being hungover were negative consequences of consuming alcohol around water (39%). One participant described that ‘Going in the water and that could make you sick or more drunk or just not feeling good’.—*P01*. Another described general feelings of discomfort associated with a hangover, ‘Not to mention the hangover that will follow the next day, anyways you'll feel like shit regardless’.—*P04*.

##### Environmental impact

Participants also described that drinking alcohol around water could have a negative impact on the environment (17%), including littering, destruction of property and damage to pools. One participant described how littering can occur, stating that ‘You have the issue of littering as well. I mean most things that people do is they'll dig a hole and bury all the bottles in the sand, or you know whatever’.—*P04*.

##### It increases the risk of drowning and injury

All participants mentioned a increased risk as a disadvantage (e.g., death, drowning or injury). One participant described that consuming alcohol around water increased likelihood of injury, ‘Well anything with the injury, so you know people getting injured or something like that. I haven't personally seen that, but I know that does happen where people get injured severely because they have been under the influence when trying to do silly things with jumping off the roof as you were saying before while consuming alcohol. They can do serious damage to their lives really’.—*P18*.

##### Inhibits my judgement

Almost three quarters of participants described that alcohol consumption negatively impacted their judgement regarding water safety and swimming ability (73%). One participant described that ‘if you're drinking too much you can obviously lose your inhibitions a little bit and do some more risky behaviour, which is a bit dangerous’.—*P19*. Another participant described the effects on coordination and judgement, stating ‘Well I mean you probably overestimate your swimming ability, you think you're much more like coordinated then you are. It also just introduces a lot of risk you know if you like suddenly get tired and you feel you could go longer than you thought you could. You might not see very clearly, you might like go in deeper than you normally would because you have more courage or bravado or whatever. You know like I said I tried to set myself a goal that I normally wouldn't. You might misjudge the conditions if the surf's too rough or something or the water's too deep, you might not consider that before you make the decision to go swimming’.—*P23*.

##### Can lead to legal punishment

Males described the legal consequences of drinking alcohol around water, such as receiving fines and getting arrested, as disadvantages related to the behaviour (13%). One participant expressed, ‘I think at most beaches that's illegal. On the boats I think you're allowed to have open containers as long as the person in charge of the boat is sober. So obviously there's the risk of all the legal side of things as well’.—*P05*.

##### Risk to one's reputation

The potential of embarrassing oneself when consuming alcohol around water was perceived as a disadvantage (21%). One participant stated that ‘This is probably the social side, not being able to swim too well and looking like a bit of a goose’—*P13*. Another stated the potential to ‘embarrass yourself in front of family members and parents, your family’.—*P04*.

### Normative beliefs

#### Approval of drinking alcohol around water

##### Approval from family

Family members, in particular parents and siblings, were among the individuals that participants identified as supporting or approving of their decisions to consume alcohol around water (52%). One participant described that ‘I don't think that my parents would worry at all if I was having a beer or a few beers’.—*P14*. Another participant described that acceptance would depend on how much was being consumed and that parents may also engage in the behaviour, stating that ‘If it's just having a few, like at my parent's, they would probably join in’.—*P03*.

##### Approval from friends

A large proportion of participants identified that friends and peers were also among those who supported or approved of the individual consuming alcohol around water (82%). It was often identified that these friends would also engage in the behaviour. One participant specifically mentioned his group of male friends, ‘Like probably the friends that I did it with, the group of guy friends, I know that the girls that I was with were less excited about it, but I mean I guess if my friends did it as well then that kind of tacit approval’.—*P23*. Another participant described reasons as to why friends would be approving, ‘Maybe like school friends or Uni [university] friends probably? Just but it's probably just because… I don't know, less mature, or like they just don't feel like being that kind of person that would say something’.—*P21*.

##### Police would not mind

Police were identified within the groups of individuals who approved of, or were indifferent towards, swimming and drinking (13%). One participant stated, ‘Oh I mean like as long as you're not drinking too heavily the cops [police] don't seem to mind’.—*P10*. Regarding participant perceptions of police opinions of drinking around water, one participant described the differences between a police officer's role and their opinions while off‐duty, stating that ‘Yeah definitely they would I think because I guess that position like they have to disapprove of anything that carries danger with it although like at the same time like I know my friends that would have a couple of beers and still go for a surf or something like that. But if they're on duty, yeah they'll definitely care but at the same time I think they also in social settings like are not that disapproving of it’.—*P21*.

#### Disapproval of drinking alcohol around water

##### Family disapproval

Although family were perceived to be approving, when asked to describe people who would disapprove of participants consuming alcohol around water, participants described family as a key objector (86%). One participant stated that disapprovers would include, ‘My mum. Probably my family probably would [disapprove]. I guess my fiancé wouldn't be too happy’.—*P23*.

##### Health and emergency services

Health and emergency services professionals were also identified by a third of participants as disapproving of the behaviour, including doctors and paramedics (34%). One participant described that doctors and other professionals would have a negative opinion towards drinking alcohol in and around water, describing that ‘My doctor, probably professionals like that would be like, “that's stupid” or something like that’.—*P23*.

##### Lifeguards

Lifeguards were also identified among those who would be disapproving of the behaviour (56%), ‘The people who would disapprove would be like the beach lifeguard that's worked twenty years on the blocks or like anybody from the you know any sort of life guarding association whatever country. They'd have big problems with it for sure’.—*P09*.

##### Police

Participants described disapproval from police towards consuming alcohol around water (52%). One participant stated, ‘Like you wouldn't get drunk around the police’.—*P21*. Although, like family, police were also believed to be approving.

### Control beliefs

#### Facilitators of drinking alcohol around water

##### Having access to alcohol

Participants identified that easily accessible alcohol facilitated alcohol consumption around water (52%). This included bottle shops (alcohol stores) being in close proximity to the water location, or pubs and clubs being near the water. One participant described ‘Well if it's easily accessible, if there's a pool bar or a bar near the beach or bottle shop near the beach or something like that, then it's probably more likely for it to happen’.—*P19*.

##### If the alcohol is not visible to authorities

Participants stated that authorities not witnessing alcohol consumption around water would make the behaviour more likely (21%). One participant described that despite visible signs and rules, ‘Like I would definitely have a drink at the beach even if it said no drinking at this beach if there was no like authority. Because if I can swim anywhere and do that I would swim there and do it’.—*P09*.

##### If it is a celebration and I am spending time with others

Participants described that alcohol consumption around water would be more likely if there was a celebration, such as a birthday party or public holiday, and friends or family were present. One participant described the likelihood of alcohol consumption and swimming would increase ‘If you're like celebrating, if something good happens you're celebrating or if the weather's warmer and a group of friends who you're comfortable with. On good occasions like birthdays or parties’.—*P17*. Another participant described an increased likelihood in a social setting with friends and family, ‘Factors probably like a get together with friends and family so having drinks with friends or family that would definitely make it a higher possibility of that occurring’.—*P18*.

##### Knowledge of water conditions gives confidence to drink

A theme was identified regarding participants' perceived knowledge about the body of water, which then was described as having an impact on their confidence and perceived skills in ensuring their own safety (26%). Thus, participants' perceived water knowledge was described as increasing their confidence to consume alcohol around water. One participant described, ‘Another reason is that I think I do have an awareness of, yeah, the weather and currents and how that shit works. And so even if I am inebriated, I can still have a somewhat basic understanding unless I'm really hammered’.—*P12*.

##### It's legal

Participants also described that if there were areas where public drinking was allowed, or if it was legal to engage in public drinking around water, this would facilitate the behaviour (26%). One participant stated, ‘I suppose like even the law as well. Like I mean if it's legal to do that like obviously you know like you want to have a few drinks you know like on a hot day you know at the beach. That would make things so much easier’.—*P16*.

##### If I was pressured

Male swimmers reported that pressure from peers would increase the likelihood of them drinking around water (17%). Rather than direct pressure on an individual person, one participant described a sense of pressure directed towards the whole group, ‘I guess it's kind of like that kind of peer pressure type kind of thing that can be… that can be definitely I think a thing? Like if someone's having one they pour a couple more it's kind of like “Oh just pass them around.” I think that's probably the biggest one I can think of yeah. As a negative yeah’.—*P06*.

##### If I saw other people doing it

Male swimmers described that if they saw others consuming alcohol around water, such as friends and family, it would increase the likelihood of them participating (21%). One participant stated that ‘Yeah I guess if I'm at a party where everyone is drinking, people are drinking I'll probably drink’.—*P13*. Another participant described that the behaviour would be more likely if other parents were also drinking, ‘I think if there was families there with their parents and stuff drinking as well then I'd probably have a couple of beers and still have a swim’.—*P14*.

##### Warm weather

Warm weather was also described as a facilitator of alcohol consumption around water (29%). One participant described ‘Yeah then I guess if it was really hot and I was really exhausted I might be more likely to want a beer or bring a beer’.*—P20*.

#### Barriers to drinking alcohol around water

##### If the alcohol is difficult to access

When asked about barriers to consuming alcohol around water, almost a third of the males described that an inability to access or afford alcohol would reduce the likelihood of engaging in the behaviour (30%). One participant described the impact of distance, stating that ‘If it's like a while away yeah to get alcohol probably won't do it’.—*P22*. Another described the impact of alcohol price, stating that alcohol consumption was less likely ‘if the only bars near the beach were super expensive’.—*P21*.

##### If I was alone

Participants described that a barrier to consuming alcohol around water would be if they were alone (26%), ‘If I was on my own it would be less likely to happen, I probably wouldn't do it’—*P18*, or if they were the only ones drinking, ‘Again, if I'm with people that wouldn't be doing that, then I wouldn't. Like if I was the only person then I wouldn't drink, yeah’.—*P08*.

##### If it wasn't safe

For some swimmers, consuming alcohol around water was described as being less likely if they assessed the environment and body of water as risky or unsafe (17%), ‘I would say safety concerns as well. So like if it's sort of body of water that I might need to actually handle myself well in. Like the beach or you know if I was to go white water rafting or something like that I probably wouldn't have a few beers before that’.—*P13*. Another participant described the safety of the environment, stating that ‘Probably having, oh having glass around the pool has definitely stopped me from drinking before. Just yeah not having glass around, which I think yeah just for safety of other people and stuff as well’.—*P14*.

##### If laws and signs were clear

Participants identified that the law and clear signage might help prevent them from consuming alcohol around water (47%). We refer to this as regulation of the law. One participant described that visual signs would stop them, ‘Like maybe if you saw more signs saying, “No drinking” or “No alcohol allowed” it might make you less likely to drink. I don't see any signs anywhere really’.*—P19*. Another participant expressed confusion surrounding laws and how this impacted whether they would drink around water, ‘I think I'm always a bit confused about the laws for drinking in public. So yeah I think… I'm always going back and forth on “yes it's illegal” and then “oh no it's not legal.” So if it's a day I think it's illegal I would not drink if there's police present but if I'm confused I think that it's fine too then I wouldn't have an issue with it. But one thing you wouldn't get drunk around the police’.—*P21*.

##### Having an ‘official’ presence around

A large proportion of the participants stated that the presence of police, hotel security or lifeguards would deter them from consuming alcohol around water (73%). This differs from the theme described as a facilitator above, as here participants referred to perceived officials enforcing the law. One participant described this as having an official kind of presence, ‘Well I guess you know if there had been an official kind of presence you know I wouldn't do it if there was a lifeguard who could see me or like a police officer who could see or something like that’.—*P23*. Another participant described that hotel security would deter them, ‘Probably like supervision if there's like hotel security or something standing there, I'm probably not going to do it’.—*P22*.

##### If you need to drive

A barrier which was described by some participants as preventing them from consuming alcohol around water was if they had to drive to and from the water location (17%), ‘Like say there was like if I had to drive or something like that I obviously wouldn't yeah’.—*P08*.

##### If I need to supervise kids

Some males identified that having the responsibility of watching over their own kids or other young family members would decrease the likelihood of them consuming alcohol around water (13%). One participant stated, ‘The things that make it harder would be I wouldn't want to do it if I had some children around that had the inability to swim. I might have one or two but I would never have past that, I would never have five or six because if one of my cousin's kids went under, if I was standing around, I'd be one of the like you'd be one of the, everybody would be running for that kind as soon as they possibly could’.—*P09*.

##### If I had something else on that day

Participants described that having other commitments on the same day would stop them from consuming alcohol around water (21%), ‘If I had something else to do like that day directly after that I had to be sober for’.—*P23*.

##### If there were kids and families present

Participants also described that the presence of children or families or being in a busy area would stop them from consuming alcohol around water (30%). One participant stated that ‘I'd be less likely to if there were kids around as well because of the riskiness’.—*P18*.

## DISCUSSION

Drowning is a significant threat to public health, with young males disproportionately impacted, especially when using alcohol around water (Hamilton, Keech, et al., 2018; Peden et al., 2021; World Health Organization, 2014). The results of the current study identified a range of salient beliefs that young males described as influencing their engagement in the risky drowning‐related behaviour of consuming alcohol around water. Thematic analysis identified various key themes in the males' descriptions about their decision to consume alcohol around water despite known risks. The themes support and extend previous research investigating alcohol consumption and participation in aquatic activities (Abercromby et al., 2020; Calverley et al., 2020, 2021). Namely, the study has identified themes which represent specific social cognitions regarding alcohol consumption around water: behavioural beliefs, normative beliefs and control beliefs. As described above, within the theory of planned behaviour, these beliefs underpin attitudes, subjective norms and perceived behavioural control, which predict intention, and in turn behaviour (Ajzen, 1991). The findings have important implications for messaging aimed at reducing alcohol consumption around water in young males. Specifically, the themes represent salient behavioural, normative and control beliefs of young males about the behaviour, which can be targeted using tailored intervention strategies to curb misuse of alcohol around water, thus saving lives. Findings from this research can inform the development of messages which can be embedded within water safety resources and public safety campaign materials. This includes using modes of delivery such as fact sheets, video infographics, social media and mainstream media campaigns which have been found to be effective for several risky aquatic activities (Hamilton et al., 2021; Hamilton, Peden, et al., 2018).

The increased risk of drowning or injury was a key disadvantage of alcohol consumption around water as identified by participants. Public safety campaigns and messaging should discourage people from consuming large amounts of alcohol around water given the well‐known risks of drowning or injury. Broadly, messaging which encourages keeping people safe while drinking around water rather than discouraging drinking altogether is recommended. This mirrors findings from recent research (Abercromby et al., 2020; Calverley et al., 2020, 2021) and evidence from this study that there are many perceived advantages of consuming alcohol around water, and high engagement despite awareness of risk. Recent research has also recommended using passive interventions such as messages on social media, radio and television to keep drowning prevention salient (Lawes et al., 2021). However, research is needed to establish the effectiveness of such approaches.

Perceptions of advantages including relaxation, enjoyment, social engagement and ‘liquid courage’ that have been associated with alcohol consumption around water can be offset by highlighting a key disadvantage, risking ones' reputation. This is important given that alcohol consumption around water generally occurs around friends and family. As such, messages could seek to create a more realistic and balanced attitude towards the behaviour by explaining that despite some perceived advantages, alcohol consumption around water can negatively impact ones' reputation and can cause embarrassment in the face of friends and family. For example, research has found that young people tend to distance themselves from friends they perceive to be problematic drinkers (de Visser et al., 2013). Additionally, opportunities to co‐design strategies with parents on how to speak to their children about alcohol around water should be explored. Teaching children and adolescents to be able to say no to alcohol in social settings can be achieved through peer‐based education (Calverley et al., 2020; Cicognani & Zani, 2011). Another potential recommendation is to conduct research to better understand the circumstances and emotions young males describe needing liquid courage to manage. Interventions may then be designed and evaluated which incorporate emotion regulation techniques or techniques which can build confidence and competence aligned to such situations, which may reduce the perceived advantage of liquid courage derived from alcohol consumption around water.

Cooling down on a hot day was also a key advantage identified by participants in this study. This is important, given the increased risk of drowning identified during the summer months, combined with known increased exposure and alcohol consumption, in particular at inland waterways (Peden et al., 2018, 2020). Therefore, consideration should be given to the timing of messages, given that warmer weather was identified as a key contributor to alcohol consumption around water. Continued messaging in the lead up to and during the warmer months of the year is recommended. Strategies such as promoting the use of non‐alcoholic refreshments to cool down around water may be explored. However, more research is needed to examine the impacts of non‐alcoholic beverages on alcohol consumption, as there is a dearth of research in this area (Miller et al., 2022).

Participants described uncertainty surrounding regulation and propensity for enforcement of alcohol consumption around water. Presence of police and other authority figures was a key barrier as identified by participants; therefore, increased enforcement presence should be considered. Clear communication of risk and regulation of alcohol consumption around water is required. Recommended strategies include clearly and consistently displaying signage and consistent regulation across areas, including designated alcohol‐free zones. Individuals in public safety roles such as police and lifeguards should be supported to consistently inform people of the risks associated with alcohol consumption around water. Efforts should also be made to ensure police and lifeguards are aware of up‐to‐date regulations. Access to alcohol was also identified as a key facilitator or barrier to alcohol consumption around water. This is an important target for change, including among policy makers and urban planners. Advocating against new alcohol stores being approved and opened in close proximity to aquatic locations should be considered (Peden et al., 2018).

While the current study provides important insights into how communication strategies and policy can be transformed to target this risky drowning‐related behaviour, several directions for future research have also been identified. Future research should test integrated health behaviour models (Hagger & Hamilton, 2020) to extend upon current findings to more fully capture the complexities of males' decisions to drink alcohol around water, especially given the ambivalence in beliefs identified. Research could also explore emergency personnel and public safety officers' experiences of dealing with individuals who have engaged in alcohol consumption around water to provide richer insights in this context. Further, investigations could be conducted with those who self‐report avoiding alcohol consumption around water to understand the alternative reasons for not consuming alcohol around water, given decisions to do and not do a behaviour are conceptually distinct and may be guided by different beliefs (Hamilton, Price, et al., 2018). Future research should also consider a co‐design approach for interventions targeting change in this risky drowning‐related behaviour (Koon et al., 2023). This could involve co‐designing and evaluating strategies for how friends can speak to each other about alcohol consumption.

### Study limitations

While the current research provides valuable insight for intervention design and further research, the results should be considered in light of some limitations. First, the qualitative research design lends itself to potential participant bias as this approach relies on participants' ability to recall their experiences and retrospective thought processes of the event, which may not be accurate (Coughlin, 1990). The data analysis approach used also comes with limitations. Theoretical thematic analysis may involve bias, where researchers' preconceptions, theoretical preferences or prior knowledge influence the selection and interpretation of themes.

Furthermore, participant responses may have been influenced by social desirability bias, given the topic of investigation (Leggett et al., 2003). The gender of the interviewer should also be considered. A female interviewer was purposively selected for this study to avoid participants assuming that an interviewer from their demographic already understands this highly prevalent behaviour and limiting their explanations. However, without directly comparing the data to data collected by a male interviewer, the true impact of this cannot be ascertained and therefore should be reflected upon as a potential limitation. Interviews were undertaken during the COVID‐19 restrictions in Australia, which could have influenced participant responses. For example, it is possible that participants had less recent experiences with behaviour than usual given these restrictions. Moreover, the efficiency of data collection and the mode of interviewing were also affected by COVID‐19; however, telephone interviewing has shown to result in openness and reduced feelings of judgement from participants (Ward et al., 2015).

## CONCLUSIONS

The current study adopted a qualitative approach to investigate and understand the knowledge, beliefs and attitudes that young Australian males hold towards consuming alcohol around water. Through theoretical thematic analyses of interviews with 23 males who had previously consumed alcohol around water, a range of salient beliefs were identified from the rich descriptions of their experiences. These beliefs can inform future research aiming to deepen our understanding of the processes underpinning drinking alcohol around water and can inform campaigns aimed at reducing young males' consumption of alcohol around water. Findings provide support for potential public safety campaigns, policy changes and intervention programs to reduce alcohol consumption around water by targeting behavioural, normative and control beliefs associated with alcohol consumption around water.

## AUTHOR CONTRIBUTIONS

**Sabryna V. Sas:** Investigation; writing – original draft; methodology; writing – review and editing; formal analysis; data curation. **Jacob J. Keech:** Conceptualization; supervision; methodology; writing – review and editing; formal analysis. **Amy E. Peden:** Conceptualization; methodology; writing – review and editing; supervision; funding acquisition. **Martin S. Hagger:** Writing – review and editing; supervision; conceptualization. **Kyra Hamilton:** Conceptualization; methodology; supervision; writing – review and editing; formal analysis; funding acquisition.

## Data Availability

The data that support the findings of this study are available on request from the corresponding author. The data are not publicly available due to privacy or ethical restrictions.
